# Multimodal Approach for Characterizing the Quality of Parent–Child Interaction: A Single Synchronization Source May Not Tell the Whole Story

**DOI:** 10.3390/biology12020241

**Published:** 2023-02-03

**Authors:** Tzipi Horowitz-Kraus, Carmel Gashri

**Affiliations:** 1 Educational Neuroimaging Group, Faculty of Education in Science and Technology, Technion, Haifa 3200003, Israel; 2 Faculty of Biomedical Engineering, Technion, Haifa 3200003, Israel; 3 Neuropsychology Department, Kennedy Krieger Institute, Baltimore, MD 21205, USA; 4 Department of Psychiatry and Behavioral Sciences, Johns Hopkins School of Medicine, Baltimore, MD 21205, USA

**Keywords:** synchronization, parent–child interaction, interaction matrix, hyperscanning

## Abstract

**Simple Summary:**

Parent–child interaction is the scaffold for future emotional and cognitive development and future well-being. As this interaction includes several domains, such as motor, speech, emotional expression, and more, characterizing the quality of parent–child interaction is often performed by a qualified person decoding it by observation. The development of computational tools allows relating to the interaction between the child and parent as synchronized data representing the correspondence between the two, focusing on brain-to-brain, voice/speech, eye contact, motor, and heart-rate signals. In this perspective, we will convey a new approach aiming to gather different sources of synchronization into one domain to reflect the quality of parent–child interaction.

**Abstract:**

The interaction between the parent and child is essential for the child’s cognitive and emotional development and sets the path for future well-being. These interactions, starting from birth, are necessary for providing the sensory stimulation the child needs in the critical time window of brain development. The characterization of parent–child interactions is traditionally performed by human decoding. This approach is considered the leading and most accurate way of characterizing the quality of these interactions. However, the development of computational tools and especially the concept of parent–child synchronization opened up an additional source of data characterizing these interactions in an objective, less human-labor manner. Such sources include brain-to-brain, voice/speech, eye contact, motor, and heart-rate synchronization. However, can a single source synchronization dataset accurately represent parent–child interaction? Will attending to the same stimulation, often resulting in a higher brain-to-brain synchronization, be considered an interactive condition? In this perspective, we will try to convey a new concept of the child–parent interaction synchronization (CHIPS) matrix, which includes the different sources of signals generated during an interaction. Such a model may assist in explaining the source of interaction alterations in the case of child/parent developmental/emotional or sensory deficits and may open up new ways of assessing interventions and changes in parent–child interactions along development. We will discuss this interaction during one of the parent–child joint activities providing opportunities for interaction, i.e., storytelling.

## 1. Introduction

### 1.1. Synchronization of Systems and Humans Is Critical for Academic Achievements

Timing is everything, and synchronization may be a specific example of that, especially the synchronization of information received from the sensory system [1]. For intact academic outcomes, such as fluent reading, a precise synchronization between the visual and auditory sensory modalities facilitated by cognitive control should take place [2]. Specifically, in the case of reading, the synchronized match between sounds and letters and then written words and their oral forms facilitates the construction of the mental lexicon allowing reading and reading comprehension [3]. However, this synchronization between modalities is not specific to academic settings; it was found that a social context enhances the ability to synchronize between sensory modalities [4]. This is in line with humans’ close monitoring of the environment to reach the optimal interaction, whereas the first interaction is between the parent and the child [5].

### 1.2. Parent–Child Interaction Is the Leading Force for Future Development and Storytelling as a Facilitator for Interaction

Human interactions shape the developing brain from early infancy [6]. Despite the numerous interactions humans experience throughout life, the most critical interaction with long-lasting effects is between parent and child [7]. Parent–child interactions are dynamic in their basis. During the interaction, the parent and the child respond to each other’s behavior [8], and this can be in multiple modalities, such as auditory, touch, motion, and visual [8,9,10]. This dynamic interaction is considered a “synchronization” as there is a temporal adaptation between the parent and the child in verbal and non-verbal communication [11]. 

A positive interaction early in life predicts the child’s language and cognitive development at 6–36 months [12,13,14,15,16] and relates to the child’s elementary school achievements [13,17]. The effect of child–parent interaction on child development and future social relationships [18] has been investigated deeply in the past five decades since Bowlby first proposed his attachment theory [19], with more studies focusing on the significance of child–mother interaction [20]. According to Bowlby’s theory, mothers who are more mentally and physically available to their child, accurately interpreting their behavior and responding consistently during their first year of life, will create a secure attachment with the child [21,22]. As the child matures, this attachment will develop into a positive approach towards self and others [23]. 

These relations between reports of maternal negative parent–child interaction and the child’s social/emotional outcomes were observed as early as infancy [24]. External factors leading to a non-sufficient parent–child interaction, such as poverty, led to similar child outcomes [25]. Additionally, atypical parent–child interactions are considered an indicator of autism syndrome disorder (ASD) [8,11], a signature of parental depression, or other mental health reasons [26]. Maternal depression is another case that may be related to non-sufficient parent–child interaction and is considered as an adverse childhood experience (ACE) [27] affecting child development [28] and may lead to long-term devastating consequences for the child (drug abuse, suicide, depression, poor health, and more [27]). Therefore, due to the above developmental outcomes, determining the quality of parent–child interaction is of high importance [29].

Parent–child storytelling is a setting allowing a unique examination of parent–child interaction and responsiveness as it involves verbal communication, eye contact, and motion [30]. We have demonstrated that a dynamic interaction between mothers and their 3–4.5-year-old children during storytelling is related to increased brain activation and functional connections in neural circuits associated with learning and language (cerebellum and left angular gurus, respectively) and cognitive control (left dorsolateral prefrontal cortex) while the child was listening to stories [30]. On the other hand, decreased child–mother interaction is associated with an overall reduced engagement of brain regions associated with cognitive/sensory abilities in the child [31]. Increased rates of maternal depression were related to decreased functional connections between visual processing regions related to imagination (or visualization) and neural circuits associated with language processing in their 4-year-old girls who listened to stories [32]. There, we suggested that while increased interaction during storytelling activities engages the child’s language and visual cortex regions (which serve as an imagination platform while listening to stories) [33], decreased interaction (or interaction quality) may be related to the inability to imagine or visualize the stories and the plot [32]. 

One method that involves verbal as well as nonverbal communication around a book and is related to enhanced child–adult interaction is dialogic reading (DR) [34,35,36,37]. DR is a storytelling method that encourages child–parent interaction using explicit instructions for engagement of knowledge, retrieval and storage of verbal information, and an ongoing monitoring process, all mediated via a constant attention orientation to the storyteller and the book [38]. Children participating in face-to-face DR training showed improved emotional [39,40] and verbal [41,42,43] interaction with their parents. We showed that 4-year-old children exposed to DR in daycare showed greater visual attention and language outcomes, including EEG correlates for these abilities [44,45].

Parental reading involves voice/motion/eye contact, while facial expression provides stimulation that keeps the child alert, attentive, and engaged while keeping up with the pace of the storytelling. In a way, this is the role of executive functions (i.e., cognitive abilities supporting learning [46]), which are also sought to synchronize the visual and auditory modalities during the child’s reading [3,47]. Per previous findings, parental reading fluency (i.e., the level of accuracy and speed of parental reading) is related to the functional connectivity of the child’s future reading network [48]. The other side of the coin is that maternal depression is related to altered functional connectivity between brain regions associated with imagery and executive functions while listening to stories at the age of 4 years [32]. Is it possible to evaluate parent–child interaction while examining all these perspectives?

### 1.3. Parent–Child Interaction Synchronization Matrix

**Figure 1:**
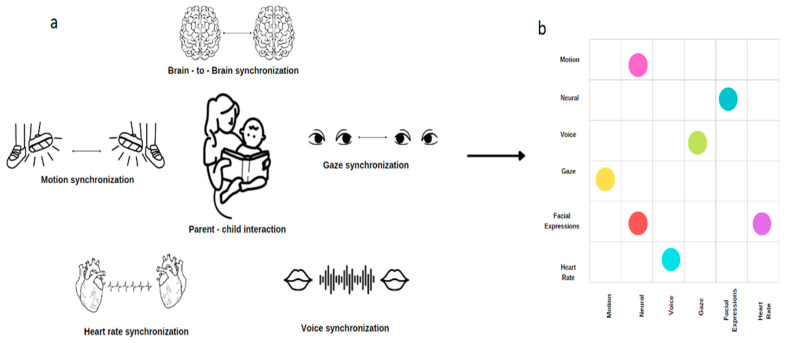
Creation of child–parent interaction synchronization (CHIPS) matrix. (**a**) Mother–child synchronization within different modalities: brain-to-brain, voice, eye contact, motion, heart rate and facial expressions synchronization; (**b**) each modality signal is taken into account in order to create a synchronization (CHIPS) matrix that shows the relation/correlation between the different signals.

Due to the determinant nature of parent–child interaction on future children’s emotional (psychological and psychopathological outcomes) [49] and cognitive/learning outcomes [50,51,52], assessing its quality is critical. One leading approach to measuring parent–child interaction is assessing the level of synchronization in several sensory modalities or different physiological signals [8] (see Figure 1a for examples of such signals). The current paper will outline the significance of each modality and the possible benefits of grouping them into one matrix. Then, a suggestion for the possible role of parent–child interaction during storytelling activities as a potential natural sensory pacemaker synchronization between parent–child will be discussed.

### 1.4. Measuring Parent–Child Interaction Using a Single Synchronization Modality Approach

Humans are engaged in social activities from birth throughout life, which led Aristotle to coin the immortal phrase, “Humans are by nature social animals” [53]. While synchronizing with others, social bonds can emerge, even with unfamiliar individuals [54], and the mental state of others can be inferred [55]. Over the years, attempts have been made to assess this synchronization, either behaviorally [56,57] or using technological tools [31,58,59]. One such approach is “Hyper-scanning”, a method that emerged in recent years to characterize interaction [60], which enables examining the neural correlates of social interactions as individuals engage in social activity [9]. Synchronization refers to the coincident rhythms of periodic oscillators due to interaction [61]. Behavioral synchronization relates to performing the same movements as others at the same time [55], whereas neural synchronization relates to the temporal coincidence of brain activities [62]. Both examples reflect the main principle of synchronization, rather than mimicry; it reflects a temporal aspect [63]. For synchronization to occur, the participants should engage in mutual attention and turn-taking for an extended period and track each other while having temporal coordination [63]. Synchronization can be measured neurobiologically [64], motorically [11], dyadically [65], mentally, and even emotionally (using facial expressions) [65]. This raises a question regarding the ability to group these interaction synchronization sources into one matrix, as well as to determine if there is a sequential pattern when examining the synchronization between these modalities. Notably, a greater parent–child synchronization, measured using different modalities, was found to be a reliable predictor for a child’s outcomes [50,51]. These studies suggest that greater parent–child synchronization during a free-play activity at the age of 4–5 years is a reliable predictor of a decrease in the child’s internalizing behavior two years after the end of the study (age 6–7 years) [51]. Another meta-analysis, including ten synchronization studies, suggested that a higher brain-to-brain synchronization at a younger age is a predictor for child self-regulation in youth [50]. Interestingly, when synchronizing two sources, although the two datasets have a similar starting point, the analysis is conducted in a “circular” manner, i.e., a gap in time has been taken into account to allowaction–reaction relations between the two individuals involved in the interaction [31,66]. However, another approach in this field in order to examine neural synchronization is using phase locking value (PLV) [67] instead of a circular manner [68]. The PLV method measures the phase difference between the datasets of the interacting dyad [67,68]. Nevertheless, as synchronization is defined as becoming coupled due to interaction, measuring synchronization using PLV may not necessarily mean that the dyad interacts [68]. In order to compare the results of studies in this field, as well as account for synchronization due to interaction, we suggest using a circular method for hyperscanning analysis.

Understanding the different synchronization modalities of individuals with communication disorders, such as ASD [9,69], can deepen the understanding of mechanisms, early markers, and treatment signatures in these populations. Here, we will overview each modality and then discuss the potential of grouping these measures into one matrix, reflecting the suggested child–parent interaction synchronization matrix (CHIPS).

### 1.5. Brain Synchronization

Hasson et al. (2012) suggested that a “brain-to-brain coupling” is a mechanism that allows the hypothetical information transfer from one individual’s brain to another to be quantified [10]. The argument was that while individuals are engaged in social interaction, there is a “wireless communication” mediated by the transmission of physical signals, generated and perceived by the individuals in an adaptive and active form, through the environment [10]. Neural synchrony can be measured both in the frequency and time domains, and synchronous activity can be detected in similar or distinct brain regions [8]. In the time domain, the EEG signal oscillations may not be perfect, and lagged phase synchrony is also considered a type of neural synchrony [8]. Most studies examine brain-to-brain synchronization using EEG focus on adults. For example, using time–frequency representation, in an EEG hyperscanning experiment, higher neural synchrony between romantic vs. stranger couples was found [70]. Additionally, adult guitar players playing together showed greater synchronization when playing a melody together vs. alone [71].

On the spatial level, functional near-infrared spectroscopy (fNIRS) studies examining adult–child interaction found higher neural synchrony between parent–child dyads in cooperation conditions [72,73]. In addition, in fNIRS experiments in parent–child dyads with children who have ASD that examined a cooperation condition, lower neural synchronization was discovered between parent–child dyads as the severity of the ASD symptoms worsened [74]. However, while using fNIRS in children has several advantages, such as the device’s tolerance to movement and portability [8], fNIRS measures hemodynamic responses; therefore, its temporal resolution is lower than EEG (several seconds compared to milliseconds in EEG) [8]. 

### 1.6. Voice Synchronization

The essence of language development is to provide a means of communication and obtain social connections between humans [75]. The human voice is embedded in the individual, can be already observed during infancy, and is considered a biological mechanism across humans and animals [75,76]. It was evident that voice synchronization, i.e., the changes in the communication style during a conversation, occurs while two individuals are interacting (specifically during a dyadic interaction) [77]. Several studies suggested that people favor voices that match their own in terms of intensity, speech rate, and frequency [78,79]. In relation to parent–child interaction, infant-directed speech was also evident when using verbal communication with an infant [80]. This communication style was suggested to gain the infant’s attention, thus improving communication and comprehension and helping the acquisition of language skills [80]. An important part of verbal synchronization is turn-taking [63]. In human–human interactions, it is important to predict the transitions between the speaker and the listener [63]. This prediction process is continuous throughout the conversation [81] and allows turn-taking alignment, which can enhance mutual understanding [82]. In a study that examined voice synchronization between mothers and infants, it was found that the process of the timing of the vocals of the mother and the infant resembles that of adult–adult interactions, suggesting that these interactions serve as the basis for the later development of a conversation pattern [83]. Mother–infant dyads who speak different languages (Spanish vs. Flemish) showed voice synchronization, which was language-independent, demonstrating a temporal adjustment of the vocals regardless of the language used [76]. 

### 1.7. Eye Contact Synchronization

A critical part of interaction is eye contact, measured using mutual eye gaze at each other or in a similar direction [84]. Eye gaze direction specifies the attention target. Therefore, eye contact between two individuals is referred to as shared attention and allows for gaining a “communicative link” during social interactions [85]. Neuroimaging studies showed that during eye contact, there is an activation of the social brain network (which includes regions such as the anterior and posterior superior temporal sulcus, amygdala, and the fusiform gyrus) [69] and language processing regions (such as the Broca and Wernicke’s areas) [86].

Additionally, in an fNIRS study, brain activity in the same brain region (prefrontal cortex; PFC) in infants and adults was found before eye contact [87,88]. This finding suggests that both the infant and the adult anticipate or drive joint behavior together [87]. These findings correspond to an EEG study showing enhanced neural synchronization in theta and alpha bands between infants and adults in mutual eye contact rather than indirect eye gaze [89]. In those with atypical development, deficits in eye contact synchronization were found (individuals with ASD and preterm children) [8,69]. 

### 1.8. Motion Synchronization

Motion synchronization is considered a performance of the same movements simultaneously, as opposed to mimicry, in which there is a time lag between the movements of the leader and the follower [88]. This type of synchronization is considered important and serves a role during social interactions [90]. There is considerable evidence for motion synchronization in humans [90], but perhaps the most known motion synchronization is gait coordination which can be observed both in laboratory experiments (when walking next to another individual on treadmills) and in natural conditions (as found by analyzing YouTube videos that show individuals who walk side-by-side) [58]. In both conditions, humans tend to synchronize their walks either by walking in phase or anti-phase with each other [58]. During conversations, there is evidence of head movement synchronization, which was also positively related to the level of conversation comprehension [91]. It was observed that those with challenges in interaction, i.e., individuals with ASD, had difficulties both intentionally and spontaneously synchronizing their movements with others, as measured by swinging a pendulum [92]. 

With respect to parent–child interaction, the mother and her child communicate in an adaptive system in the first few days after birth [93]. As an infant does not possess language skills at this age, communication is mainly non-verbal [94]. Demonstrating the specificity of this non-verbal communication to the biological parent, a weaker synchronization in motion was observed between random mother–child dyads vs. biological couples [59,95]. 

### 1.9. Facial Expression Synchronization

Facial expressions allow the delivery of emotional messages and intentions during social interaction [93,96]. Therefore, facial expression synchronization is related to the synchronization of emotional communication between individuals [96]. While watching a movie together with a positive context, people tend to synchronize their smiles even if they are unfamiliar with each other [93], a phenomenon that was absent in people with depression [97]. Facial expression synchronization is tremendously important in the parent–child relationship as it creates a secure environment and attachment patterns [98]. Additionally, to establish meaningful social connections, the social environment and the feelings of others should be correctly inferred (by accurately relating facial expressions and their corresponding emotions) [99]. This ability is called mentalizing [100] and is developed as the child grows and depends on having a mutually responsive dyadic interaction between the child and the parent [101]. Mentalizing is important as it relates to greater attachment and security between the parent and the child [102], as well as enhances the quality of social connections (more meaningful, productive, and intimate relationships) [103]. According to a study that examined facial expression synchronization in mother–child dyads in an automated way, it was found that when the child presented nonaffective facial expressions, the mother tended to smile less, and a lower level of mother–child synchrony was detected [104]. Using the Still Face Paradigm, Tronick demonstrated how infants take an active part in social interactions [105]. The Still Face experiment is divided into three parts. First, the parent and the child engage in normal interaction, which is then interrupted by the still face episode, where the parent presents a neutral facial expression following a reunion episode in which the parent and the child return to normal interaction [105]. This study demonstrated that during the still-face episode, the infants show negative effects, tend to smile less, and avert their gazes more than during the other states [105,106]. Additionally, in a study that examined facial expressions during face-to-face interactions of infants and their parents, it was found that dyads of infants and their parents who showed more depression symptoms presented more neutral and less positive facial expressions [107]. In addition, in a hyperscanning EEG study, expressing positive emotions was related to higher mother–child neural synchrony than when presenting negative emotions [108]. The abovementioned means that changes in the emotional state of the interaction can have an effect on synchronization [104].

### 1.10. Heart Rate Synchronization

Heart rate synchronization is defined as the adjustment of heart rhythms [61,109]. Evidence for the investigation of heart rate synchronization can be traced back to 1983 when a study on a 3-month-old infant showed a synchronization with the mother, father, and a stranger, with higher peaks of the spectral density of the child’s heart rate towards the mother and father vs. the stranger [110]. In a study that examined adults who were sleeping in the same bed, it was found that there is a tendency for the heart rhythms to synchronize and that there is a bidirectional interaction between the participants’ heart rhythms [109]. Romantic partners also synchronize their heart rates [111]. In relation to mother–child face-to-face interactions, in a study that examined 3-month-old infants with their mothers, both the mothers and their infants synchronized their heart rhythms within less than 1 sec lags [112].

### 1.11. Are the Parts Equal to Their Whole?

Studies examining parent–child interaction are now beginning to inspect synchronization between several modalities (i.e., brain, heart, and motor synchronization) [113]. However, the modalities were not combined on a single matrix despite findings of synchronization within different modalities [113], highlighting the importance of creating one matrix which will combine the different sources. However, one of the challenges in this field is that there is no explicit definition for interbrain neural synchronization, and it is sometimes confused with brain entrainment (the coupling of the brains’ signals due to the same external stimuli and not due to interaction) [114]. However, here, we discuss specifically real-time interaction, which is more demanding as the child’s and the parent’s responses have to be dynamic in order to achieve social coherence [115]. Therefore, as social interaction is usually composed of several modalities [116], to understand the basics of social interactions, we need to consider these modalities. 

Here, we suggest that examining each synchronization modality is not sufficient enough to define interaction, and hence, combining several modalities onto a matrix can rule out spontaneous synchronization and the entrainment of brains that are not due to interaction (see [117] for the combination of behavioral and neurobiological modalities). 

Parent–child interactions are reciprocal and include a temporal adaptation of voice, facial expression, movements, gazes, and emotional behavior since birth, thus reflecting synchronization in several modalities [11,116]. By understanding the “typical” patterns of synchronization across modalities during social interaction, we can better detect the impaired modality (or more) in individuals with atypical development suffering from communication/interaction challenges.

### 1.12. One Matrix to Include Different Synchronization Matrices?

Despite the importance of child–parent interaction to the child’s emotional [118,119], social [118], language, and cognitive development [12,13,14,15,16], we have only seen the tip of the iceberg looking at the synchronization between the two using the above modalities. It is not yet understood how the different synchronization features (e.g., brain, speech) interact with potentially new features (e.g., facial expression, motion, and heart-rate synchronization) to characterize parent–child interaction. This approach, combining the different sources into one model, may enable examining interaction in general and parent–child interaction, in particular, to an entirely new level. This goal can be achieved by creating a united, multimodal CHild–Parent Interaction Synchronization (CHIPS) matrix that can be tested against a gold-standard interaction assessment (see Figure 1 for the example). Then, examining altered interaction in atypically developing individuals will allow (a) the determination of the contribution of each modality to the lack of synchronization and decreased interaction quality or alternatively, which source contributes the most to the synchronization and (b) the determination of the effectiveness of intervention programs on the quality of interaction by examining which modality is influenced most by the intervention in different populations in an objective manner.

## 2. Clinical and Research Implications

Interactions between parents and children are extremely complicated to assess as they include several constructs. Hence, synchronization studies focusing on a single source, whether it is voice, motion, brain, heart rate, or other parent–child matrices, seem to provide only a partial understanding of this complex interaction and may overlook additional components critical for defining the quality of interaction. We may compare it to an observation of a situation from a keyhole. What we see in this very limited field of view either clearly represents the actual scenario or may be missing additional components that may even “flip” the interpretation of what we see. Such an example is the case of joint entrainment, which may result in a high level of brain-to-brain synchronization despite decreased interaction between individuals. Another example of differences in the direction of synchronization between different sources is from children with ASD showing lower motion synchronization with typical brain-to-brain synchronization with the parents [120]. We, therefore, suggest that the inclusion of as many sources into a multimodal CHIPS matrix may better reflect the quality of parent–child interaction and assist psychological decoders in the future in characterizing the quality of parent–child interaction. As the quality of parent–child interaction plays a critical role in a child’s cognitive and emotional abilities, characterizing it accurately for early identification of lower/lack of interaction or evaluating the efficiency of interaction is highly important. 

Here, we also suggested that joint storytelling, specifically DR, is a possible method with great potential to characterize parent–child interaction. Despite the promising direction this field is going towards, there are still open questions and under-investigated areas; amongst them are (1) the identity of the dyads, (2) changes in the level of synchronization, (3) the sources of data that are fed into the matrix, and (4) the child’s outcomes associated with the matrix. Regarding the identity of the dyads, it would be of interest to examine the effect of the gender of the parent: maternal vs. paternal–child interaction, especially concerning dyads and storytelling [121]. In addition, would a mother–daughter interaction be different to a mother–son interaction? Regarding the changes in the level of synchronization, it would be of interest to examine the consequences of “going out of synch”. In other words, to determine the consequences of external or internal interference in synchronization, is it recoverable, and what are the parental and child characteristics that affect this recovery (i.e., going back to synch) (see [31] for altered synchronization due to interruptions)? 

**Figure 2:**
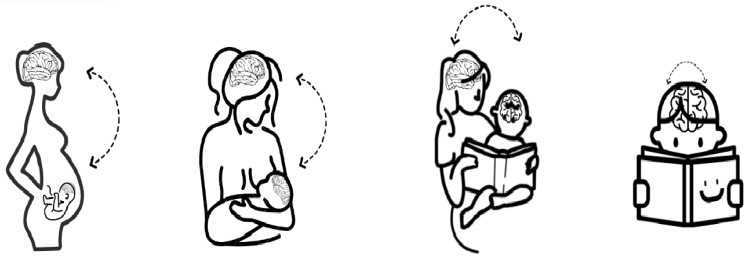
Suggested developmental progress of synchronization between the mother and child from pregnancy to reading age.

In addition, it would be interesting to examine which modality is the most dominant and affects the strength of interaction and whether it is equal for typically and atypically developing children. Lastly, there are open questions regarding the ability of these synchronization matrices to predict a child’s outcomes: is it possible that similar to the prediction of parent–child synchronization in relation to the child’s social abilities [115], parent–child joint reading and the level of interaction and synchronization in pre-reading age can shape the child’s executive functions and synchronization within the child while reading at a reading age? In other words, can multimodal synchronization during parent–child joint storytelling from birth serve as the child’s metronome for future reading fluency development? (See Figure 2 for illustration.) Does this process start even in the uterus? Additional studies are needed to verify this point.

## Data Availability

Not applicable.
